# (*E*)-3-(3,4-Dimethoxy­phen­yl)-1-(2-thien­yl)prop-2-en-1-one

**DOI:** 10.1107/S1600536808020631

**Published:** 2008-07-09

**Authors:** Hoong-Kun Fun, Samuel Robinson Jebas, P. S. Patil, S. M. Dharmaprakash

**Affiliations:** a X-ray Crystallography Unit, School of Physics, Universiti Sains Malaysia, 11800 USM, Penang, Malaysia; b Department of Studies in Physics, Mangalore University, Mangalagangotri, Mangalore 574 199, India

## Abstract

The title compound, C_15_H_14_O_3_S, has two symmetry-independent mol­ecules in the asymmetric unit with almost identical geometry. The dihedral angle between the benzene and thio­phene rings is 1.61 (11)° in one mol­ecule and 7.21 (11)° in the other. In both mol­ecules, C—H⋯O hydrogen bonds generate rings of graph-set motif *S*(5). The crystal structure is stabilized by C—H⋯O hydrogen bonds, C—H⋯π inter­actions and π–π inter­actions involving the benzene and thio­phene rings, with centroid–centroid distances of 3.5249 (13) and 3.6057 (13) Å.

## Related literature

For related literature on the biological and non-linear optical properties of chalcone derivatives, see: Agrinskaya *et al.* (1999 ▶); Chopra *et al.* (2007 ▶); Patil *et al.* (2006 ▶); Patil, Ng *et al.* (2007 ▶); Patil, Fun *et al.* (2007 ▶). For bond-length data, see: Allen *et al.* (1987 ▶). For graph-set analysis of hydrogen-bond patterns, see: Bernstein *et al.* (1995 ▶). 
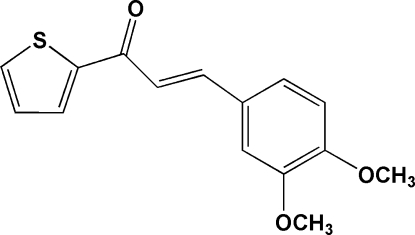


## Experimental

### 

#### Crystal data

C_15_H_14_O_3_S
*M* *_r_* = 274.32
Monoclinic, 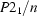

*a* = 12.1509 (3) Å
*b* = 14.3118 (3) Å
*c* = 16.3692 (4) Å
β = 106.570 (2)°
*V* = 2728.41 (11) Å^3^
*Z* = 8
Mo *K*α radiation
μ = 0.24 mm^−1^
*T* = 100.0 (1) K
0.60 × 0.17 × 0.11 mm

#### Data collection

Bruker SMART APEXII CCD area-detector diffractometer
Absorption correction: multi-scan (*SADABS*; Bruker, 2005 ▶) *T*_min_ = 0.871, *T*_max_ = 0.974
31879 measured reflections
7997 independent reflections
4723 reflections with *I* > 2σ(*I*)
*R*_int_ = 0.074

#### Refinement

*R*[*F* ^2^ > 2σ(*F* ^2^)] = 0.060
*wR*(*F* ^2^) = 0.158
*S* = 1.07
7997 reflections
347 parameters
H-atom parameters constrained
Δρ_max_ = 0.46 e Å^−3^
Δρ_min_ = −0.44 e Å^−3^

### 

Data collection: *APEX2* (Bruker, 2005 ▶); cell refinement: *APEX2*; data reduction: *SAINT* (Bruker, 2005 ▶); program(s) used to solve structure: *SHELXTL* (Sheldrick, 2008 ▶); program(s) used to refine structure: *SHELXTL*; molecular graphics: *SHELXTL*; software used to prepare material for publication: *SHELXTL* and *PLATON* (Spek, 2003 ▶).

## Supplementary Material

Crystal structure: contains datablocks global, I. DOI: 10.1107/S1600536808020631/ci2626sup1.cif

Structure factors: contains datablocks I. DOI: 10.1107/S1600536808020631/ci2626Isup2.hkl

Additional supplementary materials:  crystallographic information; 3D view; checkCIF report

## Figures and Tables

**Table 1:** Hydrogen-bond geometry (Å, °)

*D*—H⋯*A*	*D*—H	H⋯*A*	*D*⋯*A*	*D*—H⋯*A*
C7*A*—H7*AA*⋯O1*A*	0.93	2.43	2.792 (3)	103
C1*B*—H1*BA*⋯O1*A*^i^	0.93	2.36	3.261 (3)	162
C7*B*—H7*BA*⋯O1*B*	0.93	2.47	2.816 (3)	102
C14*B*—H14*F*⋯O1*B*^ii^	0.96	2.53	3.401 (3)	151
C15*A*—H15*A*⋯*Cg*1^iii^	0.96	2.92	3.616 (3)	130
C10*A*—H10*A*⋯*Cg*3^iv^	0.93	2.84	3.636 (3)	144
C3*A*—H3*AA*⋯*Cg*4	0.93	2.79	3.370 (3)	122

Symmetry codes: (i) 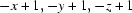; (ii) 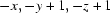; (iii) 
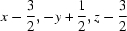
; (iv) 
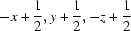
. *Cg*1, *Cg*3 and *Cg*4 are the centroids of the S1*A*/C1*A*–C4*A*, S1*B*/C1*B*–C4*B* and C8*B*–C13*B* rings, respectively.

**Fig. 1.**
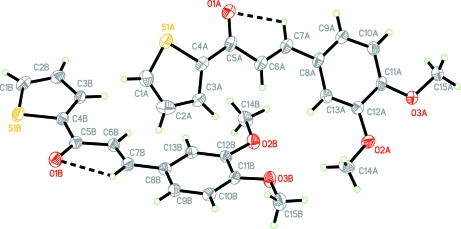
The asymmetric unit of the title compound, showing 50% probability displacement ellipsoids and the atom-numbering scheme.

**Fig. 2.**
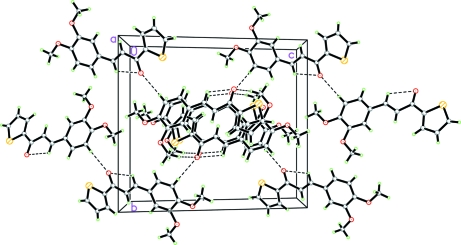
The crystal packing of the title compound, viewed along the a axis. Hydrogen bonds are shown as dashed lines.

## supplementary crystallographic information

### Comment

The synthesis and structural studies of chalcone derivatives have been of immense interest because of their biological as well as their increasingly important nonlinear optical properties (Agrinskaya *et al.*, 1999; Chopra *et al.* 2007). We have previously reported the crystal structures of D–π–A type chalcone derivatives (Patil *et al.*. 2006; Patil, Ng *et al.*, 2007; Patil, Fun *et al.*, 2007). In continuation of our interest in these compounds, we report herein the crystal structure of the title compound, (I).

There are two independent molecules (A and B) in the asymmetric unit of (I), with similar geometries (Fig. 1). The bond lengths and angles are found to have normal values (Allen *et al.*, 1987). The thiophene rings in both the molecules are planar, with a maximum deviation of 0.002 (3) Å for atom C2A and -0.007 (3) Å for atom C3B. The dihedral angle between the benzene and thiophene rings is 1.61 (11)° in molecule A and 7.21 (11)° in molecule B. In each of the independent molecule, an intramolecular C—H···O hydrogen bond generates an S(5) ring motif (Bernstein *et al.*, 1995).

The crystal structure is consolidated by weak C—H···O and C—H···π interactions (Table 1). The packing is further strengthened by π–π interactions between the S1A/C1A–C4A (centroid Cg1) and C8A–C13A (centroid Cg2) rings [Cg1···Cg2^i^ = 3.5249 (13) Å] and between the S1B/C1B–C4B (centroid Cg3) and C8B–C13B (centroid Cg4) rings [Cg3···Cg4^ii^ = 3.6057 (13) Å] [symmetry codes: (i) -*x*, 1-*y*, -*z*; (ii) -*x*, 1-*y*, -*z*].

### Experimental

The title compound was synthesized by the condensation of 3,4-dimethoxybenzaldehyde (0.01 mol, 1.66 g) with 2-acetylthiophene (0.01 mol, 1.07 ml) in methanol (60 ml) in the presence of a catalytic amount of sodium hydroxide solution (5 ml, 30%). After stirring for 6 h, the contents of the flask were poured into ice-cold water (500 ml) and left to stand for 5 h. The resulting crude solid was filtered and dried. The compound was recrystallized from acetone.

### Refinement

H atoms were positioned geometrically [C-H = 0.93-0.96 Å] and refined using a riding model, with *U*_iso_(H) = 1.2*U*_eq_(C) and 1.5U_eq_(C_methyl_). A rotating group model was used for the methyl groups.

### Crystal data

**Table:**

C_15_H_14_O_3_S	*F*_000_ = 1152
*M**_r_* = 274.32	*D*_x_ = 1.336 Mg m^−^^3^
Monoclinic, *P*2_1_/*n*	Mo *K*α radiation λ = 0.71073 Å
Hall symbol: -P 2yn	Cell parameters from 3182 reflections
*a* = 12.1509 (3) Å	θ = 2.3–22.9º
*b* = 14.3118 (3) Å	µ = 0.24 mm^−^^1^
*c* = 16.3692 (4) Å	*T* = 100.0 (1) K
β = 106.570 (2)º	Needle, white
*V* = 2728.41 (11) Å^3^	0.60 × 0.17 × 0.11 mm
*Z* = 8	

### Data collection

**Table:**

Bruker SMART APEXII CCD area-detector diffractometer	7997 independent reflections
Radiation source: fine-focus sealed tube	4723 reflections with *I* > 2σ(*I*)
Monochromator: graphite	*R*_int_ = 0.074
*T* = 100.0(1) K	θ_max_ = 30.1º
φ and ω scans	θ_min_ = 1.9º
Absorption correction: multi-scan(SADABS; Bruker, 2005)	*h* = −16→17
*T*_min_ = 0.871, *T*_max_ = 0.974	*k* = −20→20
31879 measured reflections	*l* = −23→23

### Refinement

**Table:**

Refinement on *F*^2^	Secondary atom site location: difference Fourier map
Least-squares matrix: full	Hydrogen site location: inferred from neighbouring sites
*R*[*F*^2^ > 2σ(*F*^2^)] = 0.060	H-atom parameters constrained
*wR*(*F*^2^) = 0.158	*w* = 1/[σ^2^(*F*_o_^2^) + (0.0645*P*)^2^ + 0.0849*P*] where *P* = (*F*_o_^2^ + 2*F*_c_^2^)/3
*S* = 1.07	(Δ/σ)_max_ = 0.001
7997 reflections	Δρ_max_ = 0.46 e Å^−^^3^
347 parameters	Δρ_min_ = −0.44 e Å^−^^3^
Primary atom site location: structure-invariant direct methods	Extinction correction: none

### Special details

**Table:**

Experimental. The data was collected with the Oxford Cyrosystem Cobra low-temperature attachment.
Geometry. All e.s.d.'s (except the e.s.d. in the dihedral angle between two l.s. planes) are estimated using the full covariance matrix. The cell e.s.d.'s are taken into account individually in the estimation of e.s.d.'s in distances, angles and torsion angles; correlations between e.s.d.'s in cell parameters are only used when they are defined by crystal symmetry. An approximate (isotropic) treatment of cell e.s.d.'s is used for estimating e.s.d.'s involving l.s. planes.
Refinement. Refinement of *F*^2^ against ALL reflections. The weighted *R*-factor *wR* and goodness of fit *S* are based on *F*^2^, conventional *R*-factors *R* are based on *F*, with *F* set to zero for negative *F*^2^. The threshold expression of *F*^2^ > σ(*F*^2^) is used only for calculating *R*-factors(gt) *etc*. and is not relevant to the choice of reflections for refinement. *R*-factors based on *F*^2^ are statistically about twice as large as those based on *F*, and *R*- factors based on ALL data will be even larger.

### Fractional atomic coordinates and isotropic or equivalent isotropic displacement parameters (Å^2^)

**Table:**

	*x*	*y*	*z*	*U*_iso_*/*U*_eq_	
S1A	0.30263 (5)	0.34458 (4)	0.21155 (4)	0.03061 (17)	
O1A	0.29234 (14)	0.52178 (12)	0.12000 (11)	0.0341 (4)	
O2A	−0.29137 (13)	0.76488 (11)	−0.01772 (10)	0.0271 (4)	
O3A	−0.23198 (13)	0.90679 (11)	−0.08963 (10)	0.0263 (4)	
C1A	0.2263 (2)	0.26572 (18)	0.24872 (16)	0.0347 (6)	
H1AA	0.2582	0.2124	0.2787	0.042*	
C2A	0.1135 (2)	0.28805 (18)	0.23049 (18)	0.0381 (6)	
H2AA	0.0602	0.2516	0.2470	0.046*	
C3A	0.08511 (19)	0.37196 (15)	0.18408 (14)	0.0241 (5)	
H3AA	0.0117	0.3975	0.1660	0.029*	
C4A	0.18303 (19)	0.41151 (16)	0.16895 (14)	0.0254 (5)	
C5A	0.1974 (2)	0.49958 (16)	0.12684 (14)	0.0261 (5)	
C6A	0.09567 (19)	0.56023 (16)	0.09447 (14)	0.0276 (5)	
H6AA	0.0244	0.5411	0.0987	0.033*	
C7A	0.1065 (2)	0.64255 (17)	0.05911 (15)	0.0294 (5)	
H7AA	0.1800	0.6572	0.0565	0.035*	
C8A	0.01813 (19)	0.71264 (17)	0.02396 (14)	0.0273 (5)	
C9A	0.0492 (2)	0.79240 (17)	−0.01259 (15)	0.0302 (5)	
H9AA	0.1255	0.8011	−0.0114	0.036*	
C10A	−0.03157 (19)	0.85911 (17)	−0.05077 (14)	0.0279 (5)	
H10A	−0.0096	0.9119	−0.0753	0.034*	
C11A	−0.14488 (19)	0.84704 (16)	−0.05229 (13)	0.0241 (5)	
C12A	−0.17800 (18)	0.76786 (16)	−0.01398 (14)	0.0228 (5)	
C13A	−0.09658 (19)	0.70150 (16)	0.02383 (14)	0.0248 (5)	
H13A	−0.1181	0.6492	0.0493	0.030*	
C14A	−0.3308 (2)	0.67985 (16)	0.01146 (17)	0.0321 (6)	
H14A	−0.4124	0.6830	0.0020	0.048*	
H14B	−0.2937	0.6720	0.0712	0.048*	
H14C	−0.3127	0.6278	−0.0194	0.048*	
C15A	−0.2024 (2)	0.98748 (17)	−0.13146 (16)	0.0323 (6)	
H15A	−0.2698	1.0247	−0.1547	0.048*	
H15B	−0.1719	0.9677	−0.1766	0.048*	
H15C	−0.1459	1.0238	−0.0910	0.048*	
S1B	0.37037 (5)	0.38880 (4)	0.72047 (4)	0.03122 (17)	
O1B	0.16770 (14)	0.30240 (11)	0.59734 (11)	0.0326 (4)	
O2B	−0.10219 (14)	0.60874 (11)	0.21110 (10)	0.0298 (4)	
O3B	−0.24951 (13)	0.49172 (11)	0.12281 (10)	0.0307 (4)	
C1B	0.45590 (19)	0.48496 (18)	0.73969 (15)	0.0318 (6)	
H1BA	0.5199	0.4913	0.7868	0.038*	
C2B	0.42056 (19)	0.55139 (17)	0.67877 (15)	0.0293 (5)	
H2BA	0.4578	0.6082	0.6791	0.035*	
C3B	0.32112 (18)	0.52442 (15)	0.61500 (14)	0.0236 (5)	
H3BA	0.2847	0.5621	0.5690	0.028*	
C4B	0.28323 (18)	0.43643 (15)	0.62777 (14)	0.0230 (5)	
C5B	0.18737 (18)	0.38180 (16)	0.57549 (14)	0.0240 (5)	
C6B	0.11733 (18)	0.42482 (16)	0.49650 (14)	0.0240 (5)	
H6BA	0.1316	0.4863	0.4842	0.029*	
C7B	0.03331 (19)	0.37724 (16)	0.44176 (15)	0.0262 (5)	
H7BA	0.0214	0.3165	0.4577	0.031*	
C8B	−0.04199 (18)	0.40867 (16)	0.36023 (14)	0.0236 (5)	
C9B	−0.12424 (19)	0.34745 (16)	0.31244 (14)	0.0254 (5)	
H9BA	−0.1315	0.2884	0.3340	0.030*	
C10B	−0.19590 (19)	0.37269 (16)	0.23295 (14)	0.0249 (5)	
H10B	−0.2502	0.3307	0.2018	0.030*	
C11B	−0.18609 (18)	0.45996 (16)	0.20059 (14)	0.0237 (5)	
C12B	−0.10419 (18)	0.52376 (15)	0.24885 (14)	0.0218 (5)	
C13B	−0.03375 (18)	0.49828 (16)	0.32695 (14)	0.0231 (5)	
H13B	0.0200	0.5406	0.3583	0.028*	
C14B	−0.0190 (2)	0.67467 (17)	0.25647 (16)	0.0336 (6)	
H14D	−0.0230	0.7300	0.2226	0.050*	
H14E	0.0563	0.6478	0.2683	0.050*	
H14F	−0.0345	0.6906	0.3091	0.050*	
C15B	−0.3363 (2)	0.43030 (18)	0.07416 (16)	0.0374 (6)	
H15D	−0.3730	0.4583	0.0199	0.056*	
H15E	−0.3923	0.4193	0.1043	0.056*	
H15F	−0.3020	0.3720	0.0656	0.056*	

### Atomic displacement parameters (Å^2^)

**Table:**

	*U*^11^	*U*^22^	*U*^33^	*U*^12^	*U*^13^	*U*^23^
S1A	0.0259 (3)	0.0293 (3)	0.0321 (3)	0.0024 (2)	0.0008 (2)	−0.0001 (3)
O1A	0.0258 (9)	0.0367 (10)	0.0373 (10)	0.0039 (7)	0.0047 (7)	0.0070 (8)
O2A	0.0241 (8)	0.0226 (9)	0.0342 (9)	0.0016 (7)	0.0076 (7)	0.0018 (7)
O3A	0.0254 (8)	0.0255 (9)	0.0270 (9)	0.0027 (7)	0.0060 (7)	0.0050 (7)
C1A	0.0423 (15)	0.0250 (13)	0.0364 (15)	0.0033 (11)	0.0104 (12)	−0.0002 (11)
C2A	0.0387 (15)	0.0299 (15)	0.0513 (17)	−0.0063 (11)	0.0219 (13)	−0.0096 (13)
C3A	0.0225 (11)	0.0194 (12)	0.0285 (12)	0.0018 (9)	0.0041 (9)	−0.0080 (10)
C4A	0.0240 (11)	0.0258 (13)	0.0236 (12)	0.0038 (9)	0.0021 (9)	−0.0081 (10)
C5A	0.0283 (12)	0.0251 (13)	0.0205 (11)	0.0043 (10)	0.0001 (9)	−0.0052 (10)
C6A	0.0247 (11)	0.0285 (14)	0.0261 (12)	0.0042 (10)	0.0015 (9)	−0.0030 (10)
C7A	0.0256 (12)	0.0331 (14)	0.0261 (13)	0.0038 (10)	0.0017 (10)	−0.0016 (11)
C8A	0.0245 (11)	0.0309 (14)	0.0214 (12)	0.0037 (10)	−0.0015 (9)	−0.0015 (10)
C9A	0.0237 (12)	0.0369 (15)	0.0271 (13)	0.0002 (10)	0.0027 (10)	0.0032 (11)
C10A	0.0276 (12)	0.0289 (13)	0.0251 (12)	−0.0016 (10)	0.0040 (10)	0.0036 (10)
C11A	0.0262 (11)	0.0273 (13)	0.0157 (11)	0.0042 (10)	0.0009 (9)	−0.0006 (9)
C12A	0.0229 (11)	0.0249 (12)	0.0203 (11)	0.0015 (9)	0.0054 (9)	−0.0034 (9)
C13A	0.0300 (12)	0.0215 (12)	0.0220 (12)	0.0008 (9)	0.0059 (9)	−0.0002 (9)
C14A	0.0343 (13)	0.0246 (13)	0.0423 (15)	−0.0019 (10)	0.0185 (12)	0.0014 (11)
C15A	0.0313 (13)	0.0298 (14)	0.0351 (14)	0.0028 (10)	0.0082 (11)	0.0120 (11)
S1B	0.0260 (3)	0.0321 (4)	0.0313 (3)	0.0046 (2)	0.0013 (2)	0.0055 (3)
O1B	0.0321 (9)	0.0229 (9)	0.0387 (10)	−0.0015 (7)	0.0036 (8)	0.0055 (8)
O2B	0.0342 (9)	0.0224 (9)	0.0284 (9)	−0.0067 (7)	0.0017 (7)	0.0020 (7)
O3B	0.0332 (9)	0.0256 (9)	0.0260 (9)	−0.0052 (7)	−0.0034 (7)	0.0010 (7)
C1B	0.0221 (11)	0.0390 (15)	0.0312 (13)	0.0035 (10)	0.0027 (10)	−0.0071 (12)
C2B	0.0239 (11)	0.0277 (13)	0.0344 (13)	−0.0022 (10)	0.0054 (10)	−0.0032 (11)
C3B	0.0219 (11)	0.0222 (12)	0.0243 (12)	0.0016 (9)	0.0027 (9)	0.0017 (10)
C4B	0.0227 (11)	0.0224 (12)	0.0233 (12)	0.0032 (9)	0.0058 (9)	−0.0011 (9)
C5B	0.0236 (11)	0.0230 (12)	0.0263 (12)	0.0019 (9)	0.0083 (9)	−0.0018 (10)
C6B	0.0253 (11)	0.0200 (12)	0.0253 (12)	−0.0010 (9)	0.0048 (9)	0.0014 (9)
C7B	0.0263 (12)	0.0198 (12)	0.0315 (13)	0.0013 (9)	0.0068 (10)	0.0034 (10)
C8B	0.0225 (11)	0.0257 (13)	0.0220 (11)	−0.0024 (9)	0.0053 (9)	−0.0029 (9)
C9B	0.0262 (11)	0.0233 (12)	0.0251 (12)	−0.0024 (9)	0.0048 (9)	0.0001 (10)
C10B	0.0240 (11)	0.0211 (12)	0.0274 (12)	−0.0037 (9)	0.0036 (9)	−0.0055 (10)
C11B	0.0229 (11)	0.0239 (12)	0.0218 (11)	0.0003 (9)	0.0025 (9)	−0.0007 (10)
C12B	0.0247 (11)	0.0164 (11)	0.0255 (12)	−0.0007 (9)	0.0091 (9)	0.0014 (9)
C13B	0.0229 (11)	0.0214 (12)	0.0240 (12)	−0.0037 (9)	0.0052 (9)	−0.0041 (9)
C14B	0.0400 (14)	0.0223 (13)	0.0343 (14)	−0.0124 (11)	0.0041 (11)	−0.0027 (11)
C15B	0.0413 (15)	0.0332 (15)	0.0284 (14)	−0.0105 (12)	−0.0051 (11)	0.0002 (11)

### Geometric parameters (Å, °)

**Table:**

S1A—C1A	1.681 (3)	S1B—C1B	1.699 (3)
S1A—C4A	1.714 (2)	S1B—C4B	1.724 (2)
O1A—C5A	1.232 (3)	O1B—C5B	1.235 (3)
O2A—C12A	1.362 (2)	O2B—C12B	1.368 (3)
O2A—C14A	1.439 (3)	O2B—C14B	1.426 (3)
O3A—C11A	1.362 (3)	O3B—C11B	1.364 (3)
O3A—C15A	1.439 (3)	O3B—C15B	1.429 (3)
C1A—C2A	1.355 (3)	C1B—C2B	1.356 (3)
C1A—H1AA	0.93	C1B—H1BA	0.93
C2A—C3A	1.410 (3)	C2B—C3B	1.407 (3)
C2A—H2AA	0.93	C2B—H2BA	0.93
C3A—C4A	1.402 (3)	C3B—C4B	1.377 (3)
C3A—H3AA	0.93	C3B—H3BA	0.93
C4A—C5A	1.471 (3)	C4B—C5B	1.460 (3)
C5A—C6A	1.479 (3)	C5B—C6B	1.466 (3)
C6A—C7A	1.336 (3)	C6B—C7B	1.337 (3)
C6A—H6AA	0.93	C6B—H6BA	0.93
C7A—C8A	1.462 (3)	C7B—C8B	1.457 (3)
C7A—H7AA	0.93	C7B—H7BA	0.93
C8A—C9A	1.390 (3)	C8B—C9B	1.390 (3)
C8A—C13A	1.402 (3)	C8B—C13B	1.408 (3)
C9A—C10A	1.384 (3)	C9B—C10B	1.391 (3)
C9A—H9AA	0.93	C9B—H9BA	0.93
C10A—C11A	1.381 (3)	C10B—C11B	1.375 (3)
C10A—H10A	0.93	C10B—H10B	0.93
C11A—C12A	1.408 (3)	C11B—C12B	1.414 (3)
C12A—C13A	1.384 (3)	C12B—C13B	1.370 (3)
C13A—H13A	0.93	C13B—H13B	0.93
C14A—H14A	0.96	C14B—H14D	0.96
C14A—H14B	0.96	C14B—H14E	0.96
C14A—H14C	0.96	C14B—H14F	0.96
C15A—H15A	0.96	C15B—H15D	0.96
C15A—H15B	0.96	C15B—H15E	0.96
C15A—H15C	0.96	C15B—H15F	0.96
			
C1A—S1A—C4A	91.80 (12)	C1B—S1B—C4B	91.86 (12)
C12A—O2A—C14A	116.11 (17)	C12B—O2B—C14B	117.02 (18)
C11A—O3A—C15A	116.73 (17)	C11B—O3B—C15B	116.49 (18)
C2A—C1A—S1A	112.9 (2)	C2B—C1B—S1B	112.52 (18)
C2A—C1A—H1AA	123.5	C2B—C1B—H1BA	123.7
S1A—C1A—H1AA	123.5	S1B—C1B—H1BA	123.7
C1A—C2A—C3A	113.4 (2)	C1B—C2B—C3B	112.2 (2)
C1A—C2A—H2AA	123.3	C1B—C2B—H2BA	123.9
C3A—C2A—H2AA	123.3	C3B—C2B—H2BA	123.9
C4A—C3A—C2A	110.4 (2)	C4B—C3B—C2B	113.1 (2)
C4A—C3A—H3AA	124.8	C4B—C3B—H3BA	123.5
C2A—C3A—H3AA	124.8	C2B—C3B—H3BA	123.5
C3A—C4A—C5A	130.5 (2)	C3B—C4B—C5B	130.2 (2)
C3A—C4A—S1A	111.49 (18)	C3B—C4B—S1B	110.32 (17)
C5A—C4A—S1A	118.01 (16)	C5B—C4B—S1B	119.42 (17)
O1A—C5A—C4A	120.2 (2)	O1B—C5B—C4B	120.7 (2)
O1A—C5A—C6A	121.6 (2)	O1B—C5B—C6B	122.0 (2)
C4A—C5A—C6A	118.2 (2)	C4B—C5B—C6B	117.2 (2)
C7A—C6A—C5A	119.8 (2)	C7B—C6B—C5B	121.2 (2)
C7A—C6A—H6AA	120.1	C7B—C6B—H6BA	119.4
C5A—C6A—H6AA	120.1	C5B—C6B—H6BA	119.4
C6A—C7A—C8A	128.6 (2)	C6B—C7B—C8B	128.1 (2)
C6A—C7A—H7AA	115.7	C6B—C7B—H7BA	116.0
C8A—C7A—H7AA	115.7	C8B—C7B—H7BA	116.0
C9A—C8A—C13A	119.0 (2)	C9B—C8B—C13B	118.4 (2)
C9A—C8A—C7A	118.3 (2)	C9B—C8B—C7B	118.9 (2)
C13A—C8A—C7A	122.7 (2)	C13B—C8B—C7B	122.7 (2)
C10A—C9A—C8A	121.1 (2)	C8B—C9B—C10B	121.4 (2)
C10A—C9A—H9AA	119.5	C8B—C9B—H9BA	119.3
C8A—C9A—H9AA	119.5	C10B—C9B—H9BA	119.3
C11A—C10A—C9A	119.8 (2)	C11B—C10B—C9B	119.7 (2)
C11A—C10A—H10A	120.1	C11B—C10B—H10B	120.1
C9A—C10A—H10A	120.1	C9B—C10B—H10B	120.1
O3A—C11A—C10A	124.9 (2)	O3B—C11B—C10B	125.0 (2)
O3A—C11A—C12A	114.88 (19)	O3B—C11B—C12B	115.24 (19)
C10A—C11A—C12A	120.2 (2)	C10B—C11B—C12B	119.7 (2)
O2A—C12A—C13A	125.7 (2)	O2B—C12B—C13B	124.9 (2)
O2A—C12A—C11A	114.77 (19)	O2B—C12B—C11B	114.97 (19)
C13A—C12A—C11A	119.53 (19)	C13B—C12B—C11B	120.2 (2)
C12A—C13A—C8A	120.4 (2)	C12B—C13B—C8B	120.6 (2)
C12A—C13A—H13A	119.8	C12B—C13B—H13B	119.7
C8A—C13A—H13A	119.8	C8B—C13B—H13B	119.7
O2A—C14A—H14A	109.5	O2B—C14B—H14D	109.5
O2A—C14A—H14B	109.5	O2B—C14B—H14E	109.5
H14A—C14A—H14B	109.5	H14D—C14B—H14E	109.5
O2A—C14A—H14C	109.5	O2B—C14B—H14F	109.5
H14A—C14A—H14C	109.5	H14D—C14B—H14F	109.5
H14B—C14A—H14C	109.5	H14E—C14B—H14F	109.5
O3A—C15A—H15A	109.5	O3B—C15B—H15D	109.5
O3A—C15A—H15B	109.5	O3B—C15B—H15E	109.5
H15A—C15A—H15B	109.5	H15D—C15B—H15E	109.5
O3A—C15A—H15C	109.5	O3B—C15B—H15F	109.5
H15A—C15A—H15C	109.5	H15D—C15B—H15F	109.5
H15B—C15A—H15C	109.5	H15E—C15B—H15F	109.5
			
C4A—S1A—C1A—C2A	−0.2 (2)	C4B—S1B—C1B—C2B	0.18 (19)
S1A—C1A—C2A—C3A	0.3 (3)	S1B—C1B—C2B—C3B	0.5 (3)
C1A—C2A—C3A—C4A	−0.3 (3)	C1B—C2B—C3B—C4B	−1.1 (3)
C2A—C3A—C4A—C5A	−177.2 (2)	C2B—C3B—C4B—C5B	−177.3 (2)
C2A—C3A—C4A—S1A	0.1 (2)	C2B—C3B—C4B—S1B	1.2 (2)
C1A—S1A—C4A—C3A	0.04 (18)	C1B—S1B—C4B—C3B	−0.78 (17)
C1A—S1A—C4A—C5A	177.78 (18)	C1B—S1B—C4B—C5B	177.88 (17)
C3A—C4A—C5A—O1A	−180.0 (2)	C3B—C4B—C5B—O1B	178.9 (2)
S1A—C4A—C5A—O1A	2.8 (3)	S1B—C4B—C5B—O1B	0.6 (3)
C3A—C4A—C5A—C6A	0.8 (3)	C3B—C4B—C5B—C6B	−0.5 (3)
S1A—C4A—C5A—C6A	−176.47 (16)	S1B—C4B—C5B—C6B	−178.83 (15)
O1A—C5A—C6A—C7A	−2.0 (3)	O1B—C5B—C6B—C7B	−4.4 (3)
C4A—C5A—C6A—C7A	177.2 (2)	C4B—C5B—C6B—C7B	175.0 (2)
C5A—C6A—C7A—C8A	−179.6 (2)	C5B—C6B—C7B—C8B	−178.5 (2)
C6A—C7A—C8A—C9A	−177.7 (2)	C6B—C7B—C8B—C9B	179.7 (2)
C6A—C7A—C8A—C13A	0.7 (4)	C6B—C7B—C8B—C13B	0.8 (4)
C13A—C8A—C9A—C10A	−1.7 (3)	C13B—C8B—C9B—C10B	1.1 (3)
C7A—C8A—C9A—C10A	176.7 (2)	C7B—C8B—C9B—C10B	−177.9 (2)
C8A—C9A—C10A—C11A	0.5 (4)	C8B—C9B—C10B—C11B	−0.2 (3)
C15A—O3A—C11A—C10A	1.0 (3)	C15B—O3B—C11B—C10B	3.4 (3)
C15A—O3A—C11A—C12A	−178.46 (19)	C15B—O3B—C11B—C12B	−177.40 (19)
C9A—C10A—C11A—O3A	−178.4 (2)	C9B—C10B—C11B—O3B	178.4 (2)
C9A—C10A—C11A—C12A	1.0 (3)	C9B—C10B—C11B—C12B	−0.8 (3)
C14A—O2A—C12A—C13A	−7.8 (3)	C14B—O2B—C12B—C13B	1.0 (3)
C14A—O2A—C12A—C11A	172.71 (19)	C14B—O2B—C12B—C11B	−178.52 (19)
O3A—C11A—C12A—O2A	−2.1 (3)	O3B—C11B—C12B—O2B	1.3 (3)
C10A—C11A—C12A—O2A	178.4 (2)	C10B—C11B—C12B—O2B	−179.50 (19)
O3A—C11A—C12A—C13A	178.34 (19)	O3B—C11B—C12B—C13B	−178.29 (18)
C10A—C11A—C12A—C13A	−1.1 (3)	C10B—C11B—C12B—C13B	1.0 (3)
O2A—C12A—C13A—C8A	−179.7 (2)	O2B—C12B—C13B—C8B	−179.62 (19)
C11A—C12A—C13A—C8A	−0.2 (3)	C11B—C12B—C13B—C8B	−0.1 (3)
C9A—C8A—C13A—C12A	1.6 (3)	C9B—C8B—C13B—C12B	−0.9 (3)
C7A—C8A—C13A—C12A	−176.8 (2)	C7B—C8B—C13B—C12B	178.1 (2)

### Hydrogen-bond geometry (Å, °)

**Table:**

*D*—H···*A*	*D*—H	H···*A*	*D*···*A*	*D*—H···*A*
C7A—H7AA···O1A	0.93	2.43	2.792 (3)	103
C1B—H1BA···O1A^i^	0.93	2.36	3.261 (3)	162
C7B—H7BA···O1B	0.93	2.47	2.816 (3)	102
C14B—H14F···O1B^ii^	0.96	2.53	3.401 (3)	151
C15A—H15A···Cg1^iii^	0.96	2.92	3.616 (3)	130
C10A—H10A···Cg3^iv^	0.93	2.84	3.636 (3)	144
C3A—H3AA···Cg4	0.93	2.79	3.370 (3)	122

Symmetry codes: (i) −*x*+1, −*y*+1, −*z*+1; (ii) −*x*, −*y*+1, −*z*+1; (iii) *x*−3/2, −*y*+1/2, *z*−3/2; (iv) −*x*+1/2, *y*+1/2, −*z*+1/2.
